# Implications of clonality for ageing research

**DOI:** 10.1007/s10682-017-9923-2

**Published:** 2017-11-04

**Authors:** Roberto Salguero-Gómez

**Affiliations:** 1 Department of Zoology, University of Oxford, New Radcliffe House, Radcliffe Observatory Quarter, Oxford, OX2 6GG UK; 2 Centre of Excellence in Environmental Decisions, University of Queensland, St Lucia, QLD 4072 Australia

**Keywords:** Clonal reproduction, CLO-PLA database, Demography, Fast-slow continuum, Genet, Life history strategy, Life history trait, Population ecology, Population matrix model, Senescence, Phylogenetic analyses, Ramet, Sexual reproduction

## Abstract

Senescence, an organismal performance decline with age, has historically been considered a universal phenomenon by evolutionary biologists and zoologist. Yet, increasing fertility and survival with age are nothing new to plant ecologists, among whom it is common knowledge that senescence is not universal. Recently, these two realities have come into a confrontation, begging for the rephrasing of the classical question that has led ageing research for decades: “why do we senesce?” to a more practical “what are the mechanisms by which some organisms escape from senescence?” Plants are amenable to examining this question because of their rich repertoire of life history strategies. These include the existence of permanent seed banks, vegetative dormancy and ability to produce clones, among others. Here, I use a large number of high resolution demographic models from 181 species that reflect life history strategies and their trade-offs among herbaceous perennials, succulents and shrubs measured under field conditions worldwide to examine whether senescence rates of ramets from clonal plants differ from those of whole plants reproducing either strictly sexually, or with a combination of sexual and clonal mechanisms. Contrary to the initial expectation from the mutation accumulation theory of senescence, ramets of clonal plants were more likely to exhibit senescence than those species employing sexual reproduction. I discuss why these comparisons between ramets and genets are useful, as well as its implications and future directions for ageing research.

**Electronic supplementary material:**

The online version of this article (10.1007/s10682-017-9923-2) contains supplementary material, which is available to authorized users.

## Introduction

Why do we senesce? This has been a key question in evolutionary biology that has resulted in over three million peer-review publications (keyword search “aging” in ISI Web of Knowledge, 27th September 2017). Humans are concerned with the inevitable: mortality, but also on what happens to our body’s performance before that inevitable fate. Senescence, the decline in physiological functions that ultimately scales up to declines in reproductive rates and survival rates after maturity (Hamilton 1966; Williams 1957), has until recently been considered a universal phenomenon. One of the founding parents of aging research, Hamilton, once asserted that senescence evolve always “even in the farthest reaches of almost any bizarre universe” (1996). Yet, recently, Jones et al. (2014) have reported age-specific patterns of fertility and survival that are in contradiction with the expectation for the universality of senescence. In this study, for some species like the scarlet leaved viburnum (*Viburnum furcatum*) or the red gorgonian (*Paramuricea clavata*) survival increases with age, while in others like the yellow-bellied marmot (*Marmota flaviventris*) or the freshwater crocodile (*Crocodylus johnsoni*) fertility increases with age, and in other species like the white mangrove (*Avicennia marina*) and desert tortoise (*Gopherus agassizii*) both fitness components increase with age, indicating the escape from senescence.

The question of ‘why do we senesce’ has recently undergone a paradigm shift. Research in senescence has now turned to the question ‘what are the mechanisms by which some species undergo senescence, while others escape from it?’(Baudisch and Vaupel 2012; Vaupel et al. 2004). To address this question, I argue that researchers need to consider organisms and life history strategies well beyond frequently studied groups like mammals and birds (Burger et al. 2012; Hayward et al. 2013; Jones et al. 2008; Mattison et al. 2012). Plants are ideal organisms with which to experiment and address questions in aging research due to various reasons, as reviewed by Roach (2004) and Salguero-Gómez et al. (2013). First, ‘plants are out there waiting to be counted’ as famously put by Harper (1977), the founding father of modern plant population ecology. This means that high resolution demographic information can and has been collected for hundreds of plant species (Salguero-Gómez et al. 2015; Silvertown and Charlesworth 2001). Second, no other kingdom can compete with their range of life history strategies, from plant species that produce millions of propagules per capita like orchids (Hutchings 2010) to those that produce only one (Kattge et al. 2011), or those that vary in their frequency of reproduction, from strictly semelparous (Young and Augspurger 1991) to extremely iteroparous (Eckstein et al. 2006) or those that mast (Kelly and Sork 2002; Kerkhoff 2004). Another aspect of plants that renders them particularly interesting for aging research is their wide repertoire of reproductive strategies: plant species exists where reproduction is strictly autogamous to heterogamous (Barret 2002, 2010), and from strictly sexual to mostly clonal (Brown and Eckert 2004; de Kroon and van Groenendael 1997; Eckert 2002). Examining the drivers of these life history differences is key to evolutionary ecology.

Clonal reproduction has been suggested as a likely mechanism by which modular organisms, like plants, could avoid the evolution of senescence (Finch 1990). According to the mutation accumulation theory of aging (Medawar 1952), organisms senesce because their machinery is unable to reverse deleterious mutations at a rate higher than the rate at which they occur. The plant ecophysiological literature is mined with examples of species that can compartmentalize risk such as cavitation, fungal attacks and other diseases (Orians et al. 2005; Salguero-Gómez and Casper 2010, 2011; Schenk 1999; Schenk et al. 2008; Whitaker 1946; Zanne et al. 2006). Plant clonality, whereby modules (i.e. ramet) of the individual gain a certain degree of physiological independence, but contain the same genome as the whole individual (i.e. genet), represent perhaps the epitome of risk compartmentalization. In them, it is plausible that the full excision of a relatively young ramet from the remaining genet would result in a way to escape from senescence. This is so because older individuals should, according to the mutation accumulation theory, have more deleterious mutations and thus perform worse than newly produced ramets.

Population matrix models (Caswell 2001) offer the possibility to examine some of the potential reasons as to whether and how clonality may allow plants to escape from senescence. For once, population matrix models compile in a robust manner pertinent, high-quality demographic information on the rates of survival, growth and a/sexual reproduction of individuals in populations under natural settings (Salguero-Gómez and de Kroon 2010). Robust methods have been developed to derive age-based demographic trajectories from these matrices (Caswell 2001; Caswell and Salguero-Gómez 2013; Cochran and Ellner 1992), which are often based on size or stage (Lefkovitch 1965). Due to the popularity of this demographic approach, matrix population models exist for over 1200 plant species (Salguero-Gómez et al. 2015), which allow for broad scale comparative analyses.

Here I use 181 plant species from the COMPADRE Plant Matrix Database to examine whether the ramets of clonal plant species are more likely to escape from senescence than whole plants with non-clonal abilities. I then derive a set of key life history traits (e.g. mean life expectancy, degree of iteroparity, etc.) for each species to examine how the life history strategies of clonal plants may differ from those that reproduce strictly sexually, or have the ability to reproduce both sexually and clonally. Finally, I use these life history traits in a phylogenetically-corrected multidimensional trait space to examine whether species that can reproduce clonally as well as sexually are located in a significantly different space than strictly sexually-reproducing species, and whether one can predict from these key life history traits whether clonal versus strictly sexual plants escape from or experience senescence. I discuss the value and limitations of comparing ramets and genets for future directions in ageing research.

## Materials and methods

My approach to study whether and how certain key life history traits and their underlying trade-offs may predispose clonal plant species to escape from senescence entails a series of comparative steps, both within plants that can reproduce clonally, and contrasting their results with strictly sexually reproducing plants, after the careful cross-validation of studies for the correct interpretation of the way of reproduction of each studied species.

After careful data checking and species selection (below), I calculate for each species a set of key life history traits that relate to investments on population turn over, longevity, reproduction and changes in size for each examined species (Salguero-Gómez et al. 2016). The underlying data that I use here are in the form of stage- or age-based matrix population models (MPMs), obtained from the COMPADRE Plant Matrix Database (Salguero-Gómez et al. 2015), which describe the discrete-time dynamics of the population (Caswell 2001; Lefkovitch 1965; Leslie 1945), and for which obtaining age-based demographic properties (e.g. mean life expectancy) from stage-based demographic information is possible (see Jones et al. 2014). Each element of the MPM ***A*** (Eq. 1) represents transition probabilities between/within st/ages, corresponding to the rates of survival, ageing/development/growth (matrix ***U***), and rates of sexual (***F***) and clonal (***C***) reproduction of individuals (Eq. 1). Thus, each MPM describes the life history strategy of the species/population in the conditions it was studied (Salguero-Gómez et al. 2015).

1 $${\varvec{A}} = {\varvec{U}} + {\varvec{F}} + {\varvec{C}}$$

Importantly, regardless of whether the MPM is based on age or stage (e.g. size or developmental stage), robust methods exist that allow for the calculation of (age-based) life tables, from which to obtain three key traits for senescence research: *l*_*x*_, the probability of survival from age 0 to age *x*; *m*_*x*_, the age-specific per-capita rate of sexual reproduction; and *c*_*x*_, the age-specific per-capita rate of clonal reproduction, as described in detail elsewhere (Caswell 2001; Caswell and Salguero-Gómez 2013; Cochran and Ellner 1992).

From each of these MPMs, I derive a set of life history traits (Table 1). I then analyze these complex high-dimensional data using principal component analysis (PCA) to reduce their dimensionality to manageable level of interpretable axes, and thus allow to categorize the myriad life history strategies of the several hundred organisms in the data set based on just a few axes of variation. I then use these axes of variation to predict senescence trajectories, and examine how these relationships may differ according to the mode of sexual and/or clonal reproduction. The position of species on the aforementioned principal component axes is informative of the extent to which they are constrained by trade-offs. For instance, species with high PCA scores on axes of variation of longevity and reproduction are less constrained than those with positive scores on one axis but not the other (Salguero-Gómez et al. 2016; Salguero-Gómez 2017). This approach, thus, allows to directly link trade-offs to the likelihood that a given species more likely to experience—or escape from—senescence, in the context of whether it is able to reproduce only clonally, only sexually, or using both modes of reproduction.

**Table 1:** Loadings of the life history traits grouped by their relation to turnover, and investments onto longevity, sexual/clonal reproduction, and changes in size, on the first two principal component axes

	Life history trait	Symbol	Definition	PCA 1	PCA 2
Turnover	Generation time	*T*	Number of years necessary for the individuals of a population to be fully replaced by new ones	**0.48**	0.05
Longevity	Mean life expectancy	*η*_*e*_	Mean number of years that an individual lives in the population	**0.51**	0.05
	Survival	*σ*	Mean per-capita probability of survival across stages in the life cycle of the species, weighted by the stable stage distribution (SSD)	**0.25**	**−** **0.47**
Sexual and clonal reproduction	Mature life expectancy	*L*_*α*−*ω*_	Number of years from the mean age at sexual maturity (*L*_*α*_) until the mean life expectancy (*η*_*e*_) of an individual in the population	**0.51**	0.18
	Degree of iteroparity	*S*	Spread of reproduction throughout the lifespan of the individual as quantified by Demetrius’ entropy (*S*). High/low *S* values correspond to iteroparous/semelparous populations	− 0.02	**0.60**
Sexual	Mean sexual reproduction	*φ*	Mean per-capita number of sexual recruits across stages in the life cycle of the species, weighted by the SSD	**−** **0.33**	**0.41**
Size changes	Growth	*γ*	Mean probability of transitioning forward to a larger/more developed stage in the life cycle of the species, SSD-weighted	− 0.22	**−** **0.31**
	Shrinkage	*ρ*	Mean probability of transitioning back to a smaller/less developed stage in the life cycle of the species, SSD-weighted	− 0.17	**−** **0.36**
			Percentage of explained variation	33.66%	25.05%
			Cumulative percentage of explained variation	33.66%	58.71%

Loadings in bold (≥ | ± 0.25|) indicate a relatively high contribution of the life history trait to the PCA axes

### Matrix population model and species selection criteria

COMPADRE (version 5.0.0) contains over 7500 MPMs from over 800 plant species. However, I imposed a series of selection criteria to ensure the data for the posterior analyses were directly comparable across diverse plant groups, and with a special emphasis to aspects that relate to the various ways of reproduction available to plants. The selection criteria and rationale are:

1. Included MPMs must be primitive, irreducible and non-negative (Caswell 2001) so that we could calculate a series of life history traits (Table 1).
2. Included MPMs must be from field studies representing at least 3 years of field demographic data collection, corresponding to at least two annual MPMs, in order to describe a significant window of time of the species’ life course.
3. Included MPMs must be from unmanipulated (i.e. control) conditions. MPMs constructed from artificial sites (e.g. crops) or under controlled laboratory or greenhouse conditions were not included.
4. Included MPMs must be from species modeled using an annual time-step. MPMs that used seasonal projections (Caswell 2001) were not included due to the difficulties of converting their population dynamics to an annual basis to compare with all other species’ models. For this reason, all annual species were excluded.
5. Included MPMs from the following growth forms only: epiphytes, herbaceous perennials, succulents and shrubs. Annuals (in addition to point 4 above) were excluded due to their virtual absence of clonality. Trees and palms were also excluded because in them belowground clonal connections are often not quantified demographically (R. Salguero-Gómez, pers. obs.), and because their age trajectories are drastically different compared to all other plant groups (Baudisch et al. 2013). Algae were excluded due to low sample size on a far branch in the phylogenetic tree, which would provide low statistical resolution to address my hypotheses.
6. Included MPMs describe the average population dynamics of the studied species. MPMs from contiguous temporal transitions, from multiple populations in a given study, and under unmanipulated conditions were averaged element-by-element as done elsewhere (Burns et al. 2010; Franco and Silvertown 1996, 2004).
7. Included MPMs have dimensions > 2 (i.e. with three or more stages in the life cycle) to avoid issues with quick convergence to stationary equilibrium, at which point the estimates of life history trait values and rates of senescence may be unreliable (Horvitz and Tuljapurkar 2008; Jones et al. 2014).
8. Included MPMs have stage-specific survival values < 1. Although this may seem obvious, in a small number of published models the stage-specific survival values can exceed 1 due to rounding errors or other mistakes in the original model (Salguero-Gómez et al. 2015).
9. Included MPMs never fulfilled the conditions Σ**F** = 0 and Σ**C** = 0 on the one hand, or Σ**F** = 0, Σ**C** > 0 (see Eq. 1) on the other hand, since the first case implies that no form of reproduction was recorded, and in the second case, the comparisons between ramets and genets would limit the applicability of this study, plus very few plant species exist that reproduce exclusively clonally (de Kroon and van Groenendael 1997).
10. The resulting selected species were categorized according to whether they reproduced sexually and/or clonally, and whether, being clonally reproductive, the MPM had quantified rates of clonality. Three main types of MPM emerged from this basic classification: (i) “Only sexual” MPMs, where Σ***F*** > 0 and Σ***C*** = 0, and botanical knowledge (below) revealed that the species is not clonal, (ii) “Only sexual (but clonal too)” MPMs where Σ***F*** > 0 and Σ ***C*** = 0, but botanical knowledge (below) indicated that the species is indeed clonal and thus the authors had not modelled those rates, and (iii) “*Sexual* and *clonal*” MPMs, where Σ***F*** > 0 and Σ***C*** > 0. The botanical knowledge for clonality presence/absence was obtained from the CLO-PLA database (Klimešová and de Bello 2009), a careful inspection of the original studies (See online supplementary materials), floras, and when in doubt, direct communication with the authors of the studies.
11. Included MPMs for a single species. When several studies existed for a single species, I chose the one with greater temporal, spatial and stage replication as detailed elsewhere (Salguero-Gómez 2016), or the one where the classification of an MPM into one of the three classes described in point #10 above was clearer (e.g. an MPM for a species that can reproduce clonally where the authors had quantified clonality rates explicitly was preferred over an MPM for the same species where that was not the case).

**Fig. 1:**
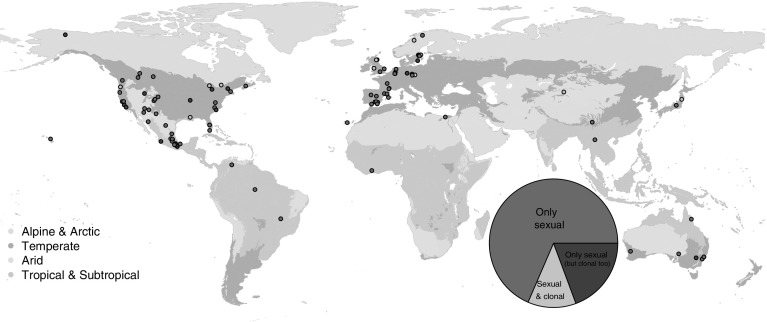
Geolocations of 118 out of the total of 181 plant species and studies here examined from the COMPADRE Plant Matrix Database (version 5.0.0), for which GPS coordinates were reported in the published studies or personally communicated. World map backgrounds showcase major habitats where the populations were studied under natural conditions. The filling color of each point corresponds to the ability of the plant species to reproduce only sexual (dark pink) or sexually as well as clonally (purple and orange). The inserted pie chart represents the proportion of studied species with population dynamics for species with only sexual reproduction (dark pink, n = 124 species), where the species can reproduce clonally, but only sexual reproduction was examined in the original study (n = 35), and species where both sexual and clonal reproduction were explicitly modelled (n = 22). (Color figure online)

The resulting set of 181 species have a broad biogeographic representation throughout all major terrestrial habitats (Fig. 1). The full citation reported in the online appendix reports both the species names as mentioned in the original publications, and their currently taxonomically accepted names as per The Plant List (http://www.theplantlist.org). The total number of species’ MPM corresponding to each mode of reproduction was: 124 species with only sexual reproduction, 35 clonal species with sexual information but no quantified rates of clonality, and 22 species with the ability for (and measurements of) sexual and clonal reproduction (Fig. 1).

I note that, although MPMs represent the demography of genets (i.e. whole organism, for only sexually reproducing species) and of ramets (for only clonally reproducing or for species that they can reproduce both sexually and clonally), their outputs are not only comparable (Baudisch et al. 2013), but *should* also be compared due to the range of questions that this analysis can raise and the very nature of macro-ecological analyses such as this one—see “Discussion”. However, the researcher must carefully consider that in these comparisons the definition of “population” changes slightly: in “only sexual” MPMs the population is most likely defined as a composite of genets (genetically different individuals), whereas in “sexual and clonal” MPMs, the definition of population happens at the ramet level, and so individuals with the same genome (ramets) *and* different genomes (ramets from different genets) are examined together in the population model. The case of “only sexual (but clonal too)” MPMs is more difficult to assess, as information on genet- versus ramet-based demography is shockingly not commonly stated in peer-reviewed publications. Despite the variation in MPM configuration, I argue that this type of comparison is necessary to test whether clonally reproducing plant species achieve their typical high longevities through having ramets that never age, or ageing ramets that live short but are replaced (Klimešová et al. 2017).

### Life history traits

**Fig. 2:**
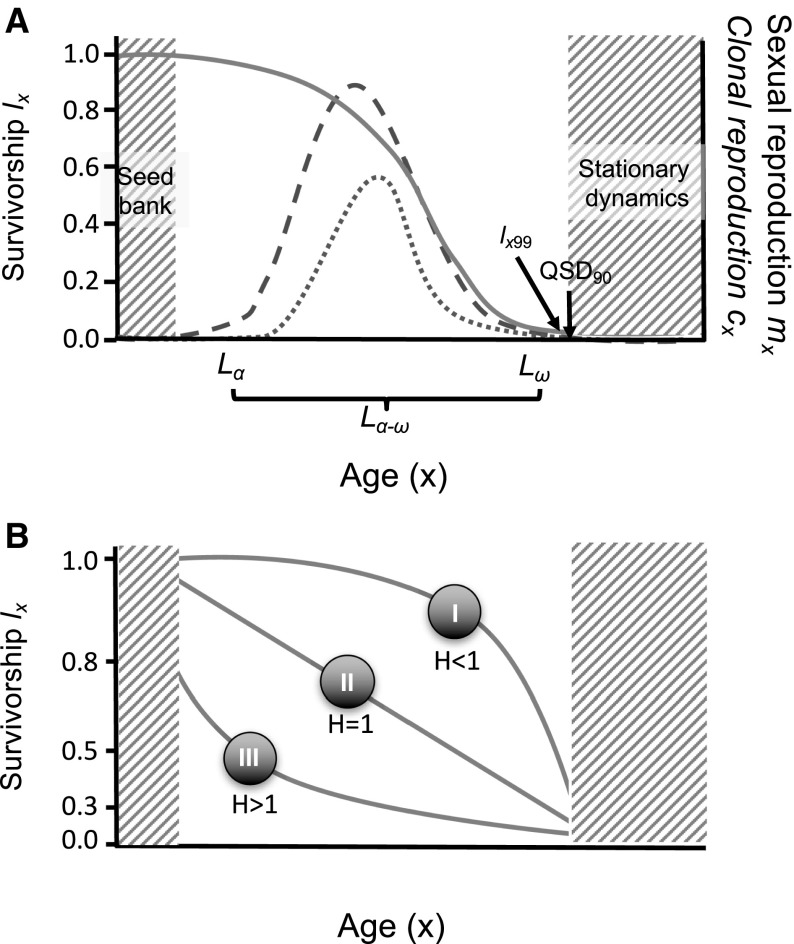
**a** An exemplified age-specific pattern for survivorship (*l*_*x*_; gray, solid line), per-capita sexual reproduction (*m*_*x*_; pink, dashed line) clonal reproduction (*c*_*x*_; blue, dotted line) obtained from a population matrix model, where age values corresponding to a permanent seed bank included in the model is left-truncated, and values corresponding stationary dynamics (e.g. mortality plateau) are right-truncated. In this study, I only considered species whose models where 99% of individuals from a cohort died (*l*_*x99*_) before the population achieved a convergence to stationary dynamics of 90% (QSD_90_). This figure also depicts how reproductive expectancy (*L*_*α*−*ω*_) was calculated, from the subtraction of time at last reproductive event (*L*_*ω*_; clonal or sexual, whichever was last), from time of first reproductive event (*L*_*α*_; clonal or sexual, whichever was first). **b** Relationship between the shape of the typical survivorship curves (in log-scale) and their relation to values of Keyfitz’ entropy (*H*). Species where most mortality happens late in life (survivorship curve type I) are characterized by *H* < 1, indicating senescence. Species where little mortality happens at advanced ages (type III) raise *H* > 1 values, indicating escape from senescence. (Color figure online)

For each species’ MPM, I derived eight key life history traits, described in more detail in Table 1. Briefly generation time (*T*) was calculated as defined in Caswell (2001, p. 126–127). Both life expectancy measures, reproductive lifetime (*L*_*α*−*ω*_) and mean life expectancy (*η*_*e*_), were calculated following methods described by Caswell (2001, p. 124). I used Demetrius entropy (Demetrius 1978) (*S*) to quantify degree of semelparity/iteroparity. To calculate this measure it is first necessary to obtain the age-specific survivorship curve (*l*_*x*_), and either/both the age-specific sexual/clonal reproduction trajectory (*m*_*x*_ */c*_*x*_; Fig. 2a). Assuming a known population growth rate λ, these can then be used to obtain *S* using Eq. 2. The calculation of *l*_*x*_ as well as *m*_*x*_ and/or *c*_*x*_ was implemented according to Caswell (2001, pp. 118–121).

2 $$S = - e^{ - log\lambda } l_{x} m_{x} log\left( {e^{ - log\lambda } l_{x} m_{x} } \right)$$

Values of *S* ≈ 0 correspond to highly-semelparous species, and large values of (*S* ≫ 0) imply high-degree of iteroparity. The vital rates of survival (*σ*), sexual reproduction (*φ*), growth (*γ*) and shrinkage (*ρ*) were averaged across the different stages (excluding seed banks) and weighted by the relative contributions of each stage at stationary equilibrium (i.e. population structure). For example, to calculate mean sexual reproduction (*φ*), I summed the values in the columns *j* of the ***F*** matrix (Eq. 1) and multiplied each *φ*_*ij*_ by the corresponding *j*th element ***w***_j_ of the stable stage distribution ***w***, calculated as the right eigenvector of ***A*** (Eq. 1; Caswell 2001).

### Life history strategies

To explore and quantify the variation exhibited in the life history traits and strategies of the 181 studies and species, I used principal component analysis (PCA). PCA is a family of multivariate statistical techniques used to examine complex data by reducing dimensionality of the data to highlight the main factors that explain the observed variation (Mardia et al. 1979). All life history traits were log-transformed in order to fulfil the assumption of normal error distribution made by PCA, and then rescaled them to mean = 0 and variance = 1. Finally, I identified and excluded outliers for each trait, which we defined as points falling outside of the 2.5th–97.5th percentile range, to aid in the display of the results. However, the results did not change qualitatively when outlying data points were not excluded (not shown).

Standard PCA approaches typically require a dataset with no missing values. In the resulting life history trait data set, ~ 23% of values were either missing due to issues in their calculation or excluded due to outlier values. Outliers here are defined as values below (above) the 2.5th (97.5th) percentiles of the mean log-transformed distribution of a given life history trait. In both situations, I used a robust protocol to impute this missing information using an iterative multilinear approach. Briefly, I employed the predictive mean matching method in the function *mice* of the R package *mice*, which uses multivariate imputation by chained equations (van Buuren and Groothuis-Oudshoorn 2011). As a check, I implemented the same protocol using only the 141 MPMs for which no data needed imputation and found that the results with imputed data were robust (not shown).

I implemented a phylogenetically-corrected PCA with the partially imputed dataset using the *phyl.pca* function in the R package *phytools* (Revell 2009, 2013). In order to explore how many axes of the PCA are sufficient to explain observed variation, I used the Kaiser criterion whereby we select axes whose associated eigenvalue is > 1 (Legendre and Legendre 2012). I then inspected the variation explained by the retained axes by obtaining the scree ranks of the PCA. Finally, I used the scores along each retained axis to quantify the life history strategies of each species’ MPM as a function of its mode of reproduction: (1) only sexual, (2) only sexual (but clonal too), and (3) sexual and clonal.

### Phylogeny

The phylogenetically-informed analysis was founded on a phylogeny created for previous demographic comparative analyses (Salguero-Gómez et al. 2015, 2016), and extended to the newly included species in the version 5.0.0 of COMPADRE using information from the Open Tree of Life (Hinchliff et al. 2016).

### Keyfitz’ entropy

Keyfitz’ entropy (*H*), also known as life table entropy, is a fitting demographic metric that quantifies heterogeneity in age at death (Keyfitz 1977; Keyfitz and Caswell 2005; Vaupel 1986) or the elasticity of life expectancy to proportional changes in age-specific mortality (Keyfitz 1977). Its calculation, using Eq. 3, requires survivorship information (i.e. *l*_*x*_) from a life table, which I first obtained for each MPM using the standard methods described by Caswell (2001). Keyfitz’ entropy is most easily understood by relating its value to the shape of the survivorship curve (*l*_*x*_) of a cohort (Fig. 2b). A so-called type I survivorship curve, resulting from a mortality rate that increases with age (i.e. senescence) has an H < 1; a Type II survivorship curve which results from a constant mortality rate (negligible senescence sensu Vaupel et al. 2004) has an *H* = 1; and a Type III survivorship curve results from a mortality rate that declines with age (negative senescence sensu Vaupel et al. 2004) with *H* > 1. Thus, the value of Keyfitz’ entropy is informative about the existence and strength of senescence.

3 $$H = \frac{{ - \mathop \int \nolimits_{0}^{\infty } { \log }(l_{x} )l_{x} dx}}{{\mathop \int \nolimits_{0}^{\infty } l_{x} dx}}$$

There are, however, two caveats to this otherwise straightforward approach. The first concerns the seed bank possessed by some plant populations, and the second concerns a mathematical artefact of MPMs. Firstly, seed bank stages are common in MPMs of plants that reproduce sexually, but pose a problem since (1) good seed bank data are scarce and (2) seed bank dynamics are likely to be only known with low certainty (Baskin and Baskin 2001). Therefore, if the MPM included seed bank stages, we ignored the dynamics resulting from the residence time in that stage. Consequently, the “start of life” in these species was considered as the time when individuals becomes *actively* established in the population. This is a common approach in comparative analysis using MPMs (Burns et al. 2010; Salguero-Gómez et al. 2016). The second potential issue arises because MPMs are typically parameterised with a stasis loop in the oldest/largest/most-developed stage (e.g. “adult survival”), which means that mortality and sexual/clonal fertility plateaus may emerge as a mathematical artefact when examining age-specific patterns (Caswell 2001; Caswell and Salguero-Gómez 2013; Horvitz and Tuljapurkar 2008). To avoid this in the calculations, only age-specific survival (*l*_*x*_) and age-specific sexual (*m*_*x*_) and clonal reproduction (*c*_*x*_) were considered until the age where the cohort approximates 90% of its stable stage distribution (the normalized right eigenvalue ***w*** of the MPM), as depicted in Fig. 2a.

### Statistical analyses

The statistical analyses involved two approaches. Firstly, I examined differences in key life history traits (Table 1) as a function of the mode of reproduction, separating plants that only reproduce sexually, only reproduce clonally, and can reproduce both sexually and clonally. I used post hoc Tukey tests to detect significant differences between groups where phylogenetic contrasts were implemented. Second, I conducted a post hoc analysis of the results of the aforementioned PCA. The Kaiser criterion determined that only the first two PCA axes should be retained, and that higher order axes could safely be ignored. I therefore fitted a two-way ANOVA model to predict Keyfitz’ entropy from the scores of these two important PCA axes, again first for all plants, and then separating for each type of MPM: “only sexual”, “only sexual (but clonal too)” and “sexual and clonal” MPMs.

## Results

The results here reported include 29 lilies (Liliopsida), and 152 flowering plants (Magnoliopsida). These species correspond to a total of 68.5% species that reproduce strictly sexually, 19.3% species that reproduce both sexually and clonally but where clonality was not quantified in the MPMs, and 12.2% species that reproduce both sexually and clonally, and where both processes were quantified in the respective MPMs (Fig. 1).

**Fig. 3:**
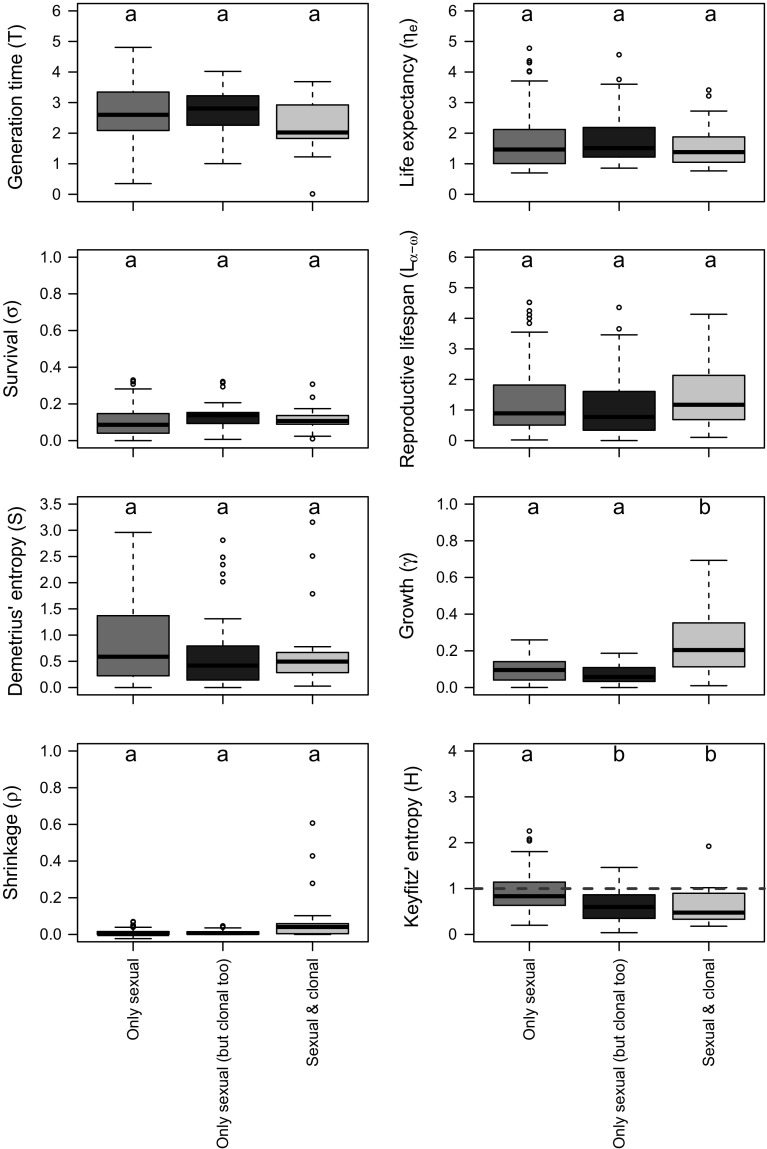
Box plots detailing differences in key life history traits for plant species that reproduce only sexually (dark pink), sexually and clonally (but where the latter was not examined in the original study; purple), or species that reproduce both sexually and clonally, and where both where both processes quantified (orange). The definition of each life history trait is detailed in Table 1. Letters within each panel correspond to post hoc Tukey test significant group differences at *P* < 0.05. Within each group, the horizontal, black, solid line represents mean, the box range the SE, the extended whiskers 95% CI, and dots represent outliers beyond the 95% CI. T, *η*_*e*_ and *L*_*α*−*ω*_ are depicted on log-scale. Species with values of Keyfitz’ entropy > 1 (horizontal dashed line) escape from senescence. (Color figure online)

Generation time (*T*), life expectancy (*η*_*e*_), rate of survival (*σ*), reproductive lifespan (*L*_*α*−*ω*_), Demetrius’ entropy (*S;* a measure of the degree of iteroparity; see Table 1), and the rate of shrinkage (*ρ*) were all not statistically significantly different among plant species with different modes of reproduction as per my classification (Fig. 3). On the other hand, the rate of growth (*γ*) of ramets in “sexual and clonal” MPMs was significantly greater than that in the “only sexual” and “only sexual (but clonal too)” MPMs (Fig. 3).

In the examined dataset, the ramets of those species with quantified “sexual and clonal” reproduction were found to systematically undergo senescence, with the exception of an outlier: *V. furcatum* (Fig. 3). In them, Keyfitz’ entropy (*H*; Fig. 2b) was significalty < 1 (*H*_*Sexual and Clonal*_ = 0.63 ± 0.08 SE; *t*_21_ = − 4.4, *P* < 0.001), which corresponds to species where mortality is more likely to occur at advanced ages (Fig. 2b). “Only sexual (but clonal too)” species also showed significant strong senescence rates (*H*_*Only sexual (but clonal too)*_ = 0.62 ± 0.06 SE; *t*_34_ = − 6.5, *P* < 0.001). These values reveal much stronger senescence rates in clonal plants than in whole plant species that reproduce strictly sexually (*H*_*Only sexual*_ = 0.90 ± 0.04). These results are visually depicted in last panel of Fig. 3, where species that can reproduce only sexually have some representative that escapes from senescence (*H* > 1), but with clonality (measured or not) ranked on average below the threshold of negligible senescence at H = 1.

**Fig. 4:**
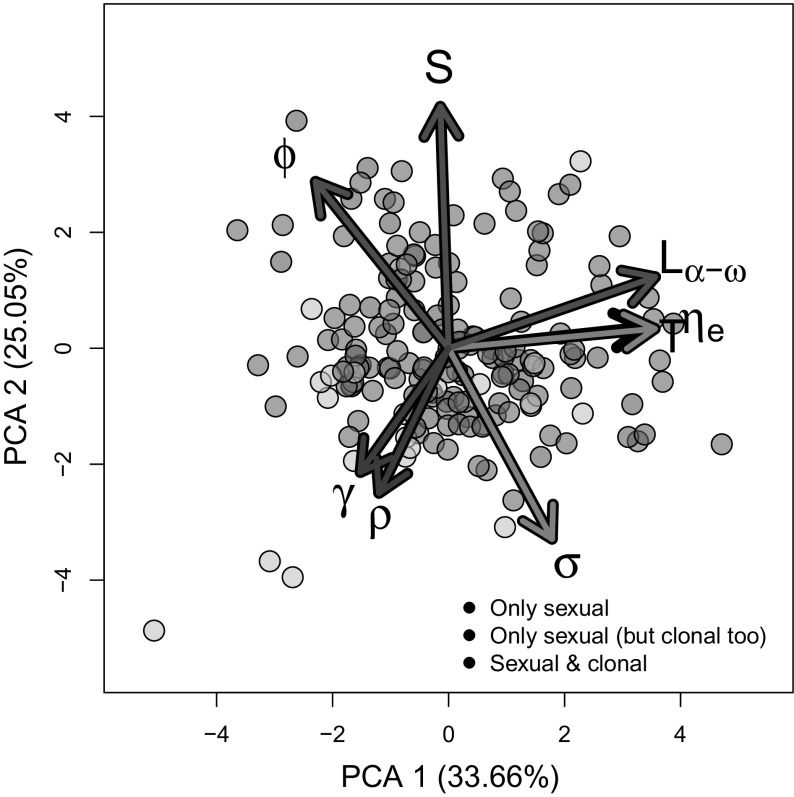
Life history trait space available to plant species as a function of their modes of reproduction. The first two axes of a principal component analyses showing how the examined 181 plant species (one per dot) are structured on a life history trait space, where the life history traits relate to population turn over (black; generation time, *T*), to reproduction, whether sexual and/or clonal (red; Demetrius’ entropy, *S*; mature life expectancy, *L*_*α*−*ω*_), to sexual reproduction (red; *ϕ*), and to changes in size (green; growth *γ* and shrinkage *ρ*). The first two axes in this PCA explain ca. 60% of the total variation in life history strategies of clonal and sexually reproducing plants as shown in Table 1. (Color figure online)

The multivariate statistical approach, using the Kaiser criterion to retain a minimum number of principal component axes to adequately explain life history strategies in the studied species, determined that only PCA 1 and 2 were necessary. Together, these axes explained ca. 60% of the total variation (Table 1). PCA 1, absorbing 33.66% of that variation, was strongly positively correlated with generation time (*T*), mean life expectancy (*η*_*e*_), and mature life expectancy (*L*_*α*−*ω*_), and negatively loaded by sexual reproduction (*φ*), implying that as one transitions from negative scores to positive scores in Fig. 4, species increase their window of reproduction and overall longevity. PCA 2, which accounted for 25.05% of the variation, was negatively correlated with growth (*γ*), shrinkage (*ρ*), and survival (*σ*), but positively loaded by the degree of iteroparity (*S*) and sexual reproduction. In other words, movement towards increasing PCA 2 scores is associated with an alteration in life history strategy from rapid oscillations in size through growth and shrinkage together with high survival and with infrequent reproduction towards a strategy of intense and frequent reproduction. Because PCA 1 is clearly strongly associated the speed of life, I henceforth refer to it as the “fast-slow continuum” (Gaillard et al. 2005; Jones et al. 2008), while I hereby refer to PCA 2 as the “reproductive strategies continuum”.

**Fig. 5:**
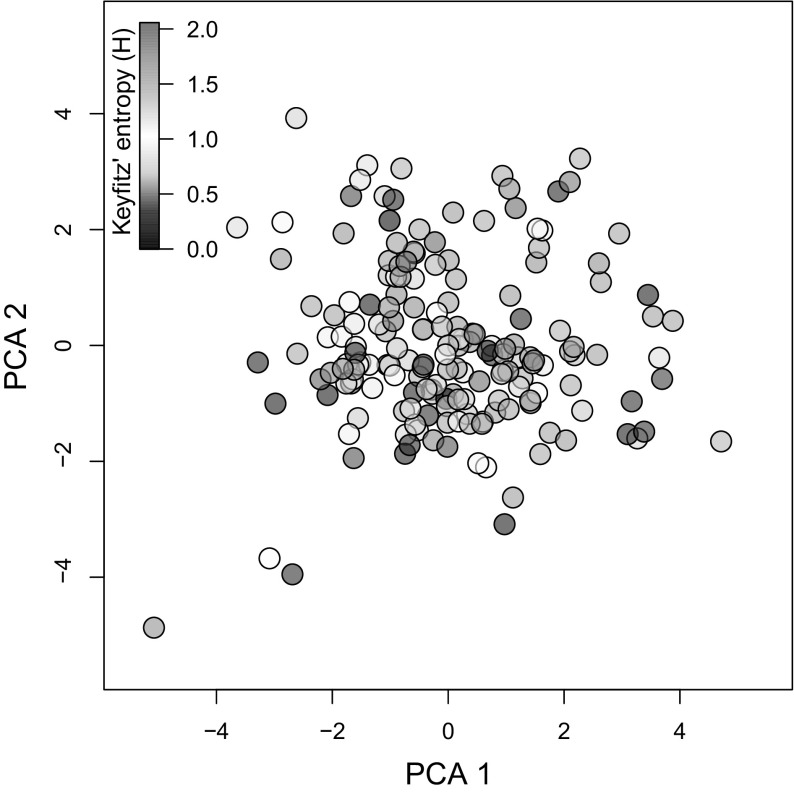
Ramets of species that reproduce sexually and clonally (orange in Fig. 4) are more likely to senesce than whole-plants that reproduce only sexually (dark pink in Fig. 4). Overall, species with the ability to reproduce clonally are located towards the bottom of the two-dimensional space defined in Fig. 4. The scores of the species along PCA 2 significantly predicts the rates of senescence, as defined by Keyfitz’ entropy, with values greater than 1 (green) corresponding to species that escape from senescence, and values < 1 (black) identifying species whose individuals typically undergo senescence. (Color figure online)

The life history strategies of the species as described by both axes, the fast-slow and the reproductive strategies continuum, predict rates of senescence of the examined plant species. When considering all the species in this analysis, as species move along the reproductive strategies continuum to increase their degree of iteroparity (*S*), and reproductive intensity, while diminishing their rates of growth and shrinkage, Keyfitz’ entropy (*H*) increases above the threshold of *H* = 1 (Table 2A), which informs on the escape from senescence (green tones in Fig. 5). No significant interactions were detected between PCA 1 and 2 with regards to their predictive power for the rate of senescence. These results remained robust when examining MPMs of “only sexual” (Table 2B), but in the case of “sexual and clonal” origins (Table 2D) only the fast-slow axis was a signficant predictor of the rates of senescence, and no significant effects were detected when evaluating separately “only sexual (but clonal too)” MPMs (Table 2C).

**Table 2:** Two-way ANOVA with the scores associated to each of the studied plant species for their alignment along the first two PCA axes of life history traits to predict values of Keyfitz’ entropy. The results are presented for (A) all species, (B) species that reproduce only sexually, (C) species that reproduce both sexually and clonally, but where the latter was not quantified in the original study, and (D) species where sexual and clonal reproduction occur and were quantified in the original studies

	Estimate	SE	*t*	*P* value
A: All plant species				
PCA 1	0.061	0.017	3.651	**<0.001**
PCA 2	0.073	0.020	3.730	**<0.001**
PCA 1 × PCA 2	0.016	0.009	1.706	0.090
B: Only sexual matrix population models (MPMs)				
PCA 1	0.054	0.022	2.469	*0.015*
PCA 2	0.080	0.027	3.005	*0.003*
PCA 1 × PCA 2	0.003	0.017	0.166	0.869
C: Only sexual (but clonal too) MPMs				
PCA 1	0.082	0.058	1.408	0.176
PCA 2	0.083	0.056	1.487	0.154
PCA 1 × PCA 2	0.017	0.018	0.971	0.345
D: Sexual and clonal MPMs				
PCA 1	0.097	0.044	2.193	*0.036*
PCA 2	0.108	0.063	1.701	0.099
PCA 1 × PCA 2	0.047	0.029	1.632	0.113

Bold: *P* < 0.001. Italic: *P* < 0.05

Whether a species had the capability of reproducing clonaly or not did not influence its placing on the two-dimensional life history strategy space described by the fast-slow continuum and the reproductive strategies continuum. A two-way ANOVA with both axes interacting with the presence/absence of clonality of the 181 examined species showed no significant main effect or interactions with clonality (Table 3A). Likewise, the role of clonality along each axes, separately, was not significant (Table 3B, C).

**Table 3:** (A) Two-way ANOVA with covariates with the scores associated to each of the studied plant species for their alignment along the first two PCA axes of life history traits (Table 1, Fig. 4) and whether or not they are clonal, with Keyfitz’ entropy as response variable, (B) two-way ANOVA with scores of PCA 1 and whether or not the species is clonal, predicting Keyfitz’ entropy, (C) two-way ANOVA with scores of PCA 2 and whether or not the species is clonal, predicting Keyfitz’ entropy

	Estimate	SE	*t*	*P* value
A: Two-way ANOVA with covariates				
PCA 1	0.062	0.019	3.294	**<0.001**
PCA 2	0.073	0.023	3.211	*0.002*
Clonality	0.082	0.099	0.831	0.407
PCA 1 × clonality	− 0.007	0.054	− 0.127	0.899
PCA 2 × clonality	− 0.007	0.057	− 0.124	0.901
B: Two-way ANOVA for PCA 1				
PCA 1	0.050	0.019	2.668	*0.008*
Clonality	0.022	0.094	0.235	0.814
PCA 1 × clonality	0.037	0.048	0.769	0.443
C. Two-way ANOVA for PCA 2				
PCA 2	0.059	0.023	2.569	*0.011*
Clonality	0.057	0.100	0.573	0.568
PCA 2 × clonality	0.036	0.052	0.702	0.483

Bold: *P* < 0.001. Italic: *P* < 0.05

## Discussion

The theory of aging via mutation accumulation predicts that senescence is universal because any organism’s biological machinery inability to revert mutations at the same rate than they appear; consequently, we senesce because we live long enough (Medawar 1952; Rose 1991). With such a mutational increase, deleterious effects accrue, resulting in the upscaling to demographic functions, whereby the probability of reproduction should decline, and the risk of mortality increase with age (Hamilton 1966). Clonality, a rather common trait in plants (Hutchings and Bradbury 1986; de Kroon and van Groenendael 1997; Klimešová and de Bello 2009; Herben et al. 2014), has been proposed as a likely mechanism by which senescence may be postponed (Finch 1990; Orive 1995), or even avoided altogether (Pedersen 1999; Shefferson et al. 2017; Vaupel et al. 2004). This is so because in clonal plans (1) age and size of the genet are typically decoupled (de Kroon et al. 2005; de Kroon and van Groenendael 1997), and so non-senescent survival and reproductive trajectories may emerge (Baudisch et al. 2013; Caswell and Salguero-Gómez 2013; Jones et al. 2014), and (2) possibly because, due to their architectural arrangements, risk spread may be physically contained (de Kroon and van Groenendael 1997; Hutchings and Bradbury 1986; Price et al. 1996; Schenk 1999; Schenk et al. 2008; Zanne et al. 2006).

The fact that clonality may be the source of eternal youth is something with which we all are –at least unconsciously– rather well versed. Crops of bananas, oranges, pineapples, and even grapes from certain vineyards in France, to mention a few, have not declined in past centuries; perhaps not by coincidence, their yields are the result of a global ramet propagation from a single or few genets (Ganapathi et al. 1992; Heloir et al. 1997). The plant literature is indeed not exception to ecological examples of how propagation and modular partitioning may slow down or even reset the biological watch. The herbaceous perennial plant *Borderea pyrenaica* does not show any signs of physiological (Morales et al. 2013) or demographic (García et al. 2011) deterioration with age; in this case, the species is known to have a highly modular design in its shoot apical meristem arrangement (García et al. 2011). Similarly, recently, Mencuccini et al. (2013) have shown a lack of physiological decline in Scots pine (*Pinus sylvestris*) module performance grafted onto older genets.

Contrary to the aforementioned predictions of life history theory, mostly based on macrovertebrates as well as on limited evidence in the Plantae kingdom, I found that the ramets of 21 out of 22 (the exception being *V. furcatum*) plant species with the ability to reproduce both clonally and sexually, and where the original studies quantified both rates, underwent senescence, as quantified by Keyfitz’ entropy (Keyfitz 1977). Consistent with this finding, I also found that all 22 clonal species where only sexual rates were quantified underwent senescence. When examining whole-plants that reproduce strictly sexually, 19% (24 species) escaped senescence, whereas 81% (100 species) showed signs of senescence. These results provide the first comparative demographic overview examining differences in age-specific performance of plants as a function of their reproductive mode, using data spanning all continents (Fig. 1) as well an unprecedented number of plant life forms. This is likely a result of life history trait trade-offs, as shown by the fact the rate of growth of ramets of clonally reproducing species is greater than that of whole-plants that reproduce strictly sexually (Fig. 3). These results, while contracting expectations from ramifications of the theory of mutation accumulation of aging, do have a basis for experimental support since mutations can accumulate on the germ line in clonal species, as shown in protozoans (Brito et al. 2010), fungi (Griffiths 1992; Taylor et al. 2015) and some long-lived clonal plants (Ally et al. 2010). Furthermore, sexual reproduction has been shown to slow down the accumulation of mutations in other modular species like fungi (Bruggeman et al. 2003).

Even though here I have shown that the majority of the ramet-based population matrix models of both sexually and clonally reproducing species underwent senescence at the ramet level, this may not necessarily preclude genets from being able to postpone or even escape senescence. Combinations of ramet births and deaths, and overlapping ramet cohorts whereby the *young* not yet senescing ramets may replace the *old*, senescing ramets, might result in a constant performance of fitness function (i.e. survival and reproduction) with age at the whole-genet level (Leopold 1975; Pedersen 1999). Regretfully, the type of data necessary to carry out a comparative demographic analysis of this sort does not yet exist for many species (but see Gardner and Mangel 1997), making the proposed approach that I have undertaken here limited for the time being. The parameterization of individuals according to more than two state variables, as would be necessary here, is not new to demography; examples with size and developmental classes (Metcalf et al. 2013; Zambrano and Salguero-Gomez 2014), size and sex (Bierzychudek 1982), or size and age (Pfister and Wang 2005) do exist in the Plantae kingdom. Applying this so-called Goodman’s matrix framework (Goodman 1969) to clonal species demography (i.e. rates of survival, changes in size, sexual and clonal reproduction, and recruitment) is feasible, but requires careful in situ tracking of dynamics of ramets within genets, which can be challenging, particularly if the ramets can expand over long distances away. However, the fact that 100% of the clonal species where only sexual reproduction was quantified underwent senescence suggests that the make-up of ramet senescence rates scales up to the whole genet. Some of the species available in COMPADRE that I did not use (due to selection criteria, see Methods above) and which did not show values of sexual reproduction (Σ***F*** = 0 in Eq. 1) may well be strictly clonally reproducing species. For them, it is possible that some of the species archived in COMPADRE determined as “strictly sexually reproducing” may in fact be able to reproduce both sexually and clonally, but that their demography may have been reported at the genet (i.e. whole-plant) level. This is an important challenge that I have only been able to overcome through the careful inspection of each source, as well as validation via communications with the authors, floras and the invaluable CLO-PLA database (Klimešová et al. 2017; Klimešová and de Bello 2009). Population ecologist would do the research community an immense favor by explicitly studying or at least depicting the biology of the species in their demographic studies, including the possibility of clonal connections using inexpensive genetic assays.

## Conclusions

Senescence, the physiological decline that results in decreased survival and reproduction of individuals with age, should no longer be treated as a universal phenomenon. Recent evidence suggests that senescence may in fact be the exception to the rule (Jones et al. 2014) in a world where *the older* may mean *the better* for fitness components performance. Plants, clonal and not alike, constitute ideal organisms with which to experiment on questions related to actuarial senescence (or lack, thereof) (Roach 2004; Salguero-Gómez et al. 2013). Here I show that ramets of clonal plants do undergo senescence, contrary to predictions from mutation accumulation theory. If genets do manage to escape senescence, the mechanisms by which this may occur, which are likely related to ramet cohort overlap and turnover, deserve further exploration. Demographic methods to accommodate ramet within genet population dynamics do exist which would allow to address this question—what is missing is the field data. This piece of research refines the search for the fountain of eternal youth by means of having discarded the ramet as the unit of selection against senescence and begging for a refocus on the generation overlap of ramets in clonal plants and modules in non-clonal species.

## Electronic supplementary material

Below is the link to the electronic supplementary material.

Supplementary material 1 (DOCX 66 kb)
