# Supplementary material for: Implications of clonality for ageing research
Source: Evol Ecol. 2017 Nov 4;32(1):9–28. doi: 10.1007/s10682-017-9923-2 (PMC6954036; doi:10.1007/s10682-017-9923-2)
Supplement: Supplementary file 1 — Supplementary material 1 (DOCX 66 kb) [file 10682_2017_9923_MOESM1_ESM.docx]

**Supplementary Online Materials**

**Implications of clonality for ageing research**

Roberto Salguero-Gómez

[rob.salguero@zoo.ox.ac.uk](mailto:rob.salguero@zoo.ox.ac.uk)

**Table S1.** Species’ Latin name, growth form (E: Epiphyte, H: Herbaceous perennial, Sh: Shrub, Su: Succulent), bibliographic sources, and basic biogeographic information from the species in the COMPADRE Plant Matrix Database version 5.0.0 used in this study. Two variables detail whether sexual reproduction (*Sexual*), and/or clonal reproduction (*Clonal*) were explicitly modelled in the matrix population models. The variable “*Clonality check*” identifies whether a species is known to be predominantly clonal or not. Country codes as per the ISO3 nomenclature employed by the United Nations.

| **Species** | **Growth form** | **Authors** | **Journal** | **DOI/ISBN** | **Publication year** | **Country** | **Sexual** | **Clonal** | **Clonality check** |
| --- | --- | --- | --- | --- | --- | --- | --- | --- | --- |
| Acacia bilimekii | Sh | Jiménez-Lobato; Valverde | J Arid Env | 10.1016/j.jaridenv.2005.07.002 | 2006 | MEX | 1 | 0 | Yes |
| Actaea spicata | H | Fröborg; Eriksson | Can J Bot | 10.1139/B03-099 | 2003 | SWE | 1 | 0 | No |
| Agrimonia eupatoria | H | Kiviniemi | Plant Ecol | *NA* | 2002 | SWE | 1 | 0 | No |
| Agropyron cristatum | H | Hansen; Wilson | J Appl Ecol | 10.1111/j.1365-2664.2006.01145.x | 2006 | CAN | 1 | 0 | No |
| Allium tricoccum | H | Nault; Gagnon | J Ecol | 10.2307/2261228 | 1993 | CAN | 0 | 1 | Yes |
| Anemone patens | H | Williams; Crone | Ecology | 10.1890/0012-9658(2006)87[3200:TIOIGO]2.0.CO;2 | 2006 | CAN | 1 | 0 | No |
| Anthericum ramosum | H | Černá; Münzbergová | PLoS ONE | 10.1371/journal.pone.0075563 | 2013 | CZE | 0 | 1 | Yes |
| Aquilegia chrysantha | H | Stubben | PhD thesis | *NA* | 2007 | USA | 1 | 0 | No |
| Ardisia elliptica | Sh | Koop; Horvitz | Ecology | 10.1890/04-1483 | 2005 | USA | 1 | 0 | No |
| Arenaria grandiflora bolosii | H | Iriondo; Albert; Giménez; Lozano; Escudero | Book | 978-84-8014-746-0 | 2009 | ESP | 1 | 0 | No |
| Argyroxiphium sandwicense | Sh | Forsyth | Oeco | 10.1007/s00442-003-1295-3 | 2003 | USA | 1 | 0 | No |
| Armeria maritima | H | Lefebvre; Chandler-Mortimer | J Appl Ecol | 10.2307/2403051 | 1984 | BEL | 1 | 0 | No |
| Armeria merinoi | H | Iriondo; Albert; Giménez; Lozano; Escudero | Book | 978-84-8014-746-0 | 2009 | ESP | 1 | 0 | No |
| Artemisia genipi | H | Marcante; Winkler; Erschbamer | Annals Bot | 10.1093/aob/mcp047 | 2009 | AUT | 1 | 0 | Yes |
| Astragalus alopecurus | H | Nicolè | PhD thesis | *NA* | 2005 | FRA | 1 | 0 | No |
| Astragalus michauxii | H | Wall; Hoffmann; Wentworth; Gray; Hohmann | Plant Ecol | 10.1007/s11258-012-0068-7 | 2012 | USA | 1 | 0 | No |
| Astragalus peckii | H | Martin; Meinke | Popul Ecol | 10.1007/s10144-012-0318-5 | 2012 | USA | 1 | 0 | No |
| Astragalus tyghensis | H | Kaye; Pyke | Ecology | 10.1890/0012-9658(2003)084[1464:TEOSTO]2.0.CO;2 | 2003 | USA | 1 | 0 | No |
| Astrophytum asterias | Su | Martinez-Avalos | PhD thesis | *NA* | 2007 | MEX | 1 | 0 | No |
| Astrophytum capricorne | Su | Bravo Espinoza | PhD thesis | *NA* | 2011 | MEX | 1 | 0 | No |
| Astrophytum ornatum | Su | Zepeda-Martinez; Manujano; Mandujano; Golubov | J Arid Env | 10.1016/j.jaridenv.2012.08.006 | 2013 | MEX | 1 | 0 | No |
| Banksia ericifolia | Sh | Bradstock; O'Connell | Aust J Ecol | 10.1111/j.1442-9993.1988.tb00999.x | 1988 | AUS | 1 | 0 | No |
| Bothriochloa ischaemum | H | Gabbard | PhD thesis | *NA* | 2003 | USA | 1 | 0 | Yes |
| Brassica insularis | H | Noel; Maurice; Mignot; Glémin; Carbonell; Justy; Guyot; Olivieri; Petit | Cons Genet | 10.1007/s10592-010-0056-1 | 2010 | FRA | 1 | 0 | No |
| Braya fernaldii | H | Squires | PhD thesis | *NA* | 2010 | CAN | 1 | 0 | No |
| Braya longii | H | Squires | PhD thesis | *NA* | 2010 | CAN | 1 | 0 | No |
| Calathea ovandensis | H | Horvitz; Schemske | Ecol Monog | 10.2307/2937136 | 1995 | MEX | 1 | 0 | No |
| Calluna vulgaris | Sh | Scandrett; Gimmingham | Vegetatio | 10.1007/BF00036515 | 1989 | GBR | 0 | 1 | Yes |
| Calochortus albus | H | Fiedler | J Ecol | 10.2307/2260308 | 1987 | USA | 1 | 0 | Yes |
| Calochortus obispoensis | H | Fiedler | J Ecol | 10.2307/2260308 | 1987 | USA | 1 | 0 | Yes |
| Calochortus pulchellus | H | Fiedler | J Ecol | 10.2307/2260308 | 1987 | USA | 1 | 0 | Yes |
| Calochortus tiburonensis | H | Fiedler | J Ecol | 10.2307/2260308 | 1987 | USA | 1 | 0 | Yes |
| Carduus nutans | H | Jongejans; Sheppard; Shea | J Appl Ecol | 10.1111/j.1365-2664.2006.01228.x | 2006 | FRA | 1 | 0 | No |
| Carlina vulgaris | H | Lofgren; Eriksson; Lehtila | Ann Bot Fen | *NA* | 2000 | SWE | 1 | 0 | No |
| Carnegiea gigantea | Su | Steenbergh; Lowe | Ecology | 10.2307/1933696 | 1969 | USA | 1 | 0 | No |
| Cassia nemophila | Sh | Silander | Oecologia | 10.1007/BF00379524 | 1983 | AUS | 1 | 0 | No |
| Centaurea jacea | H | Jongejans; de Kroon | J Ecol | 10.1111/j.1365-2745.2005.01003.x | 2005 | NLD | 0 | 1 | Yes |
| Centaurea podospermifolia | H | Emery; Gross | J Appl Ecol | 10.1111/j.1365-2664.2004.00990.x | 2005 | USA | 0 | 1 | Yes |
| Cephalocereus senilis | Su | Cedillo Castillo | MSc thesis | *NA* | 2007 | MEX | 1 | 0 | No |
| Chaerophyllum aureum | H | Magda; Duru; Theau | Weed Sci | 10.1614/P2202-067 | 2004 | FRA | 1 | 0 | No |
| Chamaecrista lineata keyensis | H | Liu; Menges; Quintana-Ascencio | Ecol Appl | 10.1890/03-5382 | 2005 | USA | 1 | 0 | No |
| Cirsium acaule | H | Münzbergová | Am J Bot | 10.3732/ajb.92.12.1987 | 2005 | CZE | 1 | 0 | Yes |
| Cirsium dissectum | H | Jongejans; de Vere; de Kroon | Plant Ecol | 10.1007/s11258-008-9397-y | 2008 | NLD | 0 | 1 | Yes |
| Cirsium palustre | H | Ramula | Acta Oeco | 10.1016/j.actao.2007.11.005 | 2008 | SWE | 1 | 0 | No |
| Cirsium perplexans | H | Dodge | PhD thesis | *NA* | 2005 | USA | 1 | 0 | No |
| Cirsium scariosum | H | Dodge | PhD thesis | *NA* | 2005 | USA | 1 | 0 | No |
| Cirsium tracyi | H | Dodge | PhD thesis | *NA* | 2005 | USA | 1 | 0 | Yes |
| Cirsium vulgare | H | Bullock; Hill; Silvertown | J Ecol | 10.2307/2261390 | 1994 | GBR | 1 | 0 | No |
| Cleistesiopsis bifaria | H | Wells; Willems | Book | 10.2307/2261246 | 1991 | USA | 1 | 0 | Yes |
| Cleistesiopsis divaricata | H | Wells; Willems | Book | 10.2307/2261246 | 1991 | USA | 1 | 0 | Yes |
| Cochlearia bavarica | H | Abs | Folia Geobot | 10.1007/BF02803075 | 1999 | DEU | 1 | 0 | No |
| Cochlearia pyrenaica | H | Abs | Folia Geobot | 10.1007/BF02803075 | 1999 | DEU | 1 | 0 | No |
| Coespeletia spicata | Su | Silva; Trevisan; Estrada; Monosterio | Global Ecol Biogeogr | 10.1046/j.1365-2699.2000.00187.x | 2000 | VEN | 1 | 0 | No |
| Coespeletia timotensis | Su | Silva; Trevisan; Estrada; Monosterio | Global Ecol Biogeogr | 10.1046/j.1365-2699.2000.00187.x | 2000 | VEN | 1 | 0 | No |
| Colchicum autumnale | H | Winter; Jung; Eckstein; Otte; Donath; Kriechbaum | J Appl Ecol | 10.1111/1365-2664.12217 | 2014 | AUT | 1 | 0 | Yes |
| Cynoglossum officinale | H | Boorman; Fuller | New Phyto | 10.1111/j.1469-8137.1984.tb03596.x | 1984 | GBR | 1 | 0 | No |
| Cypripedium parviflorum | H | Shefferson; Warren II.; Pulliam | J Ecol | 10.1111/1365-2745.12281 | 2014 | *NA* | 1 | 0 | Yes |
| Cytisus scoparius | Sh | Neubert; Parker | Risk Anal | 10.1111/j.0272-4332.2004.00481.x | 2004 | USA | 1 | 0 | No |
| Daphne rodriguezii | Sh | Rodriguez-Perez; Traveset | Oikos | 10.1111/j.1600-0706.2011.19946.x | 2012 | ESP | 1 | 0 | No |
| Daucus carota | H | Verkaar; Schenkeveld | New Phyto | 10.1111/j.1469-8137.1984.tb04155.x | 1984 | NLD | 1 | 0 | No |
| Dicerandra frutescens | H | Menges; Quintana-Ascencio; Weekley; Gaoue | Biol Cons | 10.1016/j.biocon.2005.08.002 | 2006 | USA | 1 | 0 | No |
| Dioscorea chouardii | H | Garcia | Cons Biol | 10.1016/S0006-3207(01)00113-6 | 2003 | ESP | 1 | 0 | No |
| Dipsacus fullonum | H | Werner; Caswell | Ecology | 10.2307/1936930 | 1977 | USA | 1 | 0 | No |
| Disporum sessile | H | Kawano; Takada; Nakayama; Hiratsuka | Book | *NA* | 1987 | JPN | 1 | 0 | Yes |
| Draba asterophora | H | Putnam | PhD thesis | *NA* | 2013 | USA | 1 | 0 | No |
| Dracocephalum austriacum | H | Andrello | PhD thesis | *NA* | 2010 | FRA | 1 | 0 | No |
| Echeveria longissima | Su | Martorell | Popul Ecol | 10.1007/s10144-012-0307-8 | 2007 | MEX | 0 | 1 | Yes |
| Echinocactus platyacanthus | Su | Jiménez-Sierra; Mandujano; Eguiarte | Biol Cons | 10.1016/j.biocon.2006.10.038 | 2007 | MEX | 1 | 0 | No |
| Echinospartum ibericum algibicum | H | Iriondo; Albert; Giménez; Lozano; Escudero | Book | 978-84-8014-746-0 | 2009 | ESP | 1 | 0 | No |
| Eriogonum longifolium gnaphalifolium | H | Satterthwaite; Menges; Quintana-Ascencio | Ecol Appl | 10.1890/1051-0761(2002)012[1672:ASBPVI]2.0.CO;2 | 2002 | USA | 1 | 0 | No |
| Eryngium alpinum | H | Andrello; Bizoux; Barbet-Massin; Gaudeul; Nicolè; Till-Bottraud | Biol Cons | 10.1016/j.biocon.2011.12.012 | 2012 | FRA | 1 | 0 | No |
| Eryngium cuneifolium | H | Menges; Quintana-Ascencio | Ecol Monog | 10.1890/03-4029 | 2004 | USA | 1 | 0 | No |
| Erythronium japonicum | H | Kawano; Takada; Nakayama; Hiratsuka | Book | *NA* | 1987 | JPN | 1 | 0 | No |
| Escobaria robbinsorum | Su | Schmalzel; Reichenbacher; Rutman | Madrono | *NA* | 1995 | USA | 1 | 0 | No |
| Escontria chiotilla | Su | Ortega-Baes | PhD thesis | *NA* | 2001 | MEX | 1 | 0 | No |
| Eupatorium perfoliatum | H | Byers; Meagher | Ecol Appl | 10.1890/1051-0761(1997)007[0519:ACODCI]2.0.CO;2 | 1997 | USA | 0 | 1 | Yes |
| Eupatorium resinosum | H | Byers; Meagher | Ecol Appl | 10.1890/1051-0761(1997)007[0519:ACODCI]2.0.CO;2 | 1997 | USA | 0 | 1 | Yes |
| Fritillaria meleagris | H | Zhang; Hytteborn | Hol Ecol | 10.1111/j.1600-0587.1985.tb01174.x | 1985 | SWE | 1 | 0 | Yes |
| Gardenia actinocarpa | Sh | Osunkoya | Biol Cons | 10.1016/S0006-3207(02)00417-2 | 2003 | AUS | 1 | 0 | No |
| Gentiana pneumonanthe | H | Oostermeijer; Brugman; de Boer; den Nijs | J Ecol | 10.2307/2261351 | 1996 | NLD | 1 | 0 | No |
| Gentianella campestris | H | Lennartsson; Oostermeijer | J Ecol | 10.1046/j.1365-2745.2001.00566.x | 2001 | SWE | 1 | 0 | No |
| Geum rivale | H | Kiviniemi | Plant Ecol | *NA* | 2002 | SWE | 1 | 0 | Yes |
| Helenium virginicum | H | Adams; Marsh; Knox | Biol Cons | 10.1016/j.biocon.2005.02.001 | 2005 | USA | 1 | 0 | No |
| Helianthemum juliae | Sh | Marrero-Gómez; Oostermeijer; Carqué-Álamo; Bañares-Baudet | Biol Cons | 10.1016/j.biocon.2007.01.010 | 2007 | ESP | 1 | 0 | No |
| Helianthemum polygonoides | H | Iriondo; Albert; Gimenez; Lozano; Escudero | Book | 978-84-8014-746-0 | 2009 | ESP | 1 | 0 | No |
| Heliconia acuminata | H | Bruna | Ecology | 10.1890/0012-9658(2003)084[0932:APPIFH]2.0.CO;2 | 2003 | BRA | 1 | 0 | No |
| Heracleum mantegazzianum | H | Nehrbass; Winkler; Pergl; Perglová; Pyšek | Pers Plant Ecol Evol Syst | 10.1016/j.ppees.2005.11.001 | 2006 | *NA* | 1 | 0 | No |
| Heteropogon contortus | H | O'Connor | J Appl Ecol | 10.2307/2404276 | 1993 | ZAF | 1 | 0 | No |
| Hieracium floribundum | H | Thomas; Dale | Can J Bot | 10.1139/b75-331 | 1975 | CAN | 1 | 0 | Yes |
| Himantoglossum hircinum | H | Pfeifer; Wiegand; Heinrich; Jetschke | J Appl Ecol | 10.1111/j.1365-2664.2006.01148.x | 2006 | DEU | 1 | 0 | Yes |
| Hyparrhenia diplandra | H | Garnier; Dajoz | J Ecol | 10.1890/0012-9658(2001)082[1720:ESOALV]2.0.CO;2 | 2001 | CIV | 1 | 0 | Yes |
| Hypericum cumulicola | H | Quintana-Ascencio; Menges; Weekley | Cons Biol | 10.1046/j.1523-1739.2003.01431.x | 2003 | USA | 1 | 0 | No |
| Hypochaeris radicata | H | Jongejans; de Kroon | J Ecol | 10.1111/j.1365-2745.2005.01003.x | 2005 | NLD | 0 | 1 | Yes |
| Ipomopsis tenuituba | H | Campbell; Waser | Am Nat | 10.1086/510758 | 2007 | USA | 1 | 0 | No |
| Iris germanica | H | Burns; Pardini; Schutzenhofer; Chung; Seidler; Knight | Ecology | 10.1890/12-1310.1 | 2013 | USA | 1 | 0 | Yes |
| Isatis tinctoria | H | Farah; Tanaka; West | Weed Sci | *NA* | 1988 | USA | 1 | 0 | Yes |
| Jacobaea vulgaris | H | Forbes | Weed Res | 10.1111/j.1365-3180.1977.tb00498.x | 1977 | GBR | 1 | 0 | Yes |
| Jurinea fontqueri | H | Iriondo; Albert; Gimenez; Lozano; Escudero | Book | 978-84-8014-746-0 | 2009 | ESP | 1 | 0 | No |
| Kosteletzkya pentacarpos | H | Pino; Picó; Roa | Bot J Lin Soc | 10.1111/j.1095-8339.2007.00628.x | 2007 | ESP | 1 | 0 | No |
| Lactuca virosa | H | Boorman; Fuller | New Phyto | 10.1111/j.1469-8137.1984.tb03596.x | 1984 | GBR | 1 | 0 | No |
| Lantana camara | Sh | Osunkoya; Perrett; Fernando; Clark; Raghu | Popul Ecol | 10.1007/s10144-013-0364-7 | 2013 | AUS | 1 | 0 | Yes |
| Lathyrus vernus | H | Ehrlen | J Ecol | *NA* | 1995 | SWE | 1 | 0 | No |
| Lechea deckertii | H | Maliakal Witt | PhD thesis | *NA* | 2004 | USA | 1 | 0 | No |
| Limonium carolinianum | H | Baltzer; Reekie; Hewlin; Taylor; Boates | Can J Bot | 10.1139/b02-070 | 2002 | CAN | 1 | 0 | No |
| Limonium delicatulum | H | Hegazy | J Appl Ecol | 10.2307/2404462 | 1992 | EGY | 1 | 0 | No |
| Limonium erectum | H | Iriondo; Albert; Giménez; Lozano; Escudero | Book | 978-84-8014-746-0 | 2009 | ESP | 1 | 0 | No |
| Limonium geronense | H | Iriondo; Albert; Giménez; Lozano; Escudero | Book | 978-84-8014-746-0 | 2009 | ESP | 1 | 0 | No |
| Limonium malacitanum | H | Iriondo; Albert; Giménez; Lozano; Escudero | Book | 978-84-8014-746-0 | 2009 | ESP | 1 | 0 | No |
| Linum catharticum | H | Verkaar; Schenkeveld | New Phyto | 10.1111/j.1469-8137.1984.tb04155.x | 1984 | NLD | 1 | 0 | No |
| Linum flavum | H | Münzbergová | Plant Biology | 10.1111/plb.12007 | 2013 | CZE | 1 | 0 | No |
| Linum tenuifolium | H | Münzbergová | Plant Biology | 10.1111/plb.12007 | 2013 | CZE | 1 | 0 | No |
| Lithospermum ruderale | H | Bricker; Maron | Ecology | 10.1890/11-0948.1 | 2012 | USA | 1 | 0 | No |
| Lobularia maritima | H | Picó; de Kroon; Retano | Ecology | 10.1890/0012-9658(2002)083[1991:AEFAFS]2.0.CO;2 | 2002 | ESP | 1 | 0 | No |
| Lomatium cookii | H | Kaye; Pyke | Ecology | 10.1890/0012-9658(2003)084[1464:TEOSTO]2.0.CO;2 | 2003 | USA | 1 | 0 | No |
| Lophophora diffusa | Su | Dias Segura | MSc thesis | *NA* | 2013 | MEX | 1 | 0 | No |
| Lupinus arboreus | Sh | Kauffman; Maron | Am Nat | 10.1086/507877 | 2006 | USA | 1 | 0 | No |
| Lupinus lepidus | H | Bishop | PhD thesis | *NA* | 1996 | USA | 1 | 0 | No |
| Lupinus tidestromii | H | Dangremond; Knight | Ecology | 10.1890/09-0418.1 | 2010 | USA | 1 | 0 | No |
| Mammillaria hernandezii | Su | Rodriguez Ortega | PhD thesis | *NA* | 2008 | MEX | 1 | 0 | No |
| Mammillaria huitzilopochtli | Su | Flores-Martinez; Manzanero-Medino; Golubov; Montaña; Mandujano | Plant Ecol | 10.1007/s11258-010-9737-6 | 2010 | MEX | 1 | 0 | No |
| Mammillaria magnimamma | Su | Valverde; Quijas; Lopez-Villavicencio; Castillo | Plant Ecol | 10.1023/B:VEGE.0000021662.78634.de | 2004 | MEX | 1 | 0 | No |
| Mammillaria pectinifera | Su | Valverde; Zavala-Hurtado | J Arid Env | 10.1016/j.jaridenv.2005.06.001 | 2006 | MEX | 1 | 0 | No |
| Mammillaria solisioides | Su | Rodriguez Ortega | PhD thesis | *NA* | 2008 | MEX | 1 | 0 | No |
| Miconia albicans | Sh | Hoffmann | Ecology | 10.2307/177080 | 1999 | BRA | 1 | 0 | No |
| Mimulus cardinalis | H | Angert | Ecology | 10.1890/0012-9658(2006)87[2014:DOCAMP]2.0.CO;2 | 2006 | USA | 1 | 0 | Yes |
| Mimulus lewisii | H | Angert | Ecology | 10.1890/0012-9658(2006)87[2014:DOCAMP]2.0.CO;2 | 2006 | USA | 1 | 0 | Yes |
| Molinia caerulea | H | Jacquemyn; Brys; Neubert | Ecol Appl | 10.1890/04-1762 | 2005 | BEL | 1 | 0 | Yes |
| Narcissus pseudonarcissus | H | Barkham | J Ecol | 10.2307/2259425 | 1980 | GBR | 1 | 0 | Yes |
| Neobuxbaumia polylopha | Su | Arroyo-Cosultchi; Golubov; Mandujano | Acta Oecol | 10.1016/j.actao.2016.01.006 | 2016 | MEX | 1 | 0 | No |
| Oenothera deltoides | H | Thomson | Cons Biol | 10.1111/j.1523-1739.2005.004108.x | 2005 | USA | 1 | 0 | No |
| Opuntia macrocentra | Su | Mandujano; Golubov; Huenneke | Popul Ecol | 10.1007/s10144-006-0032-2 | 2007 | MEX | 0 | 1 | Yes |
| Orchis purpurea | H | Jacquemyns; Brys; Jongejans | Ecology | 10.1890/08-2321.1 | 2010 | BEL | 1 | 0 | No |
| Pachycereus pecten-aboriginum | Su | Morales-Romero; Godinez-Alvarez; Campo-Alves; Molino-Freaner | J Arid Env | 10.1016/j.jaridenv.2011.09.005 | 2012 | MEX | 1 | 0 | No |
| Paeonia officinalis | H | Andrieu; Freville; Besnord; Vaudey; Gauthier; Thompson; Debussche | Popul Ecol | 10.1007/s10144-012-0346-1 | 2013 | FRA | 1 | 0 | No |
| Panax quinquefolius | H | Van de Voort; McGraw | Biol Cons | 10.1016/j.biocon.2006.01.010 | 2006 | USA | 1 | 0 | Yes |
| Paronychia pulvinata | H | Forbis; Doak | Am J Bot | 10.3732/ajb.91.7.1147 | 2004 | USA | 1 | 0 | No |
| Pedicularis furbishiae | H | Menges | Cons Biol | 10.1111/j.1523-1739.1990.tb00267.x | 1990 | USA | 1 | 0 | No |
| Periandra mediterranea | H | Hoffmann; Solbrig | Forest Ecol Manag | 10.1016/S0378-1127(02)00566-2 | 2003 | BRA | 1 | 0 | No |
| Persoonia bargoensis | Sh | McKenna | PhD thesis | *NA* | 2007 | AUS | 1 | 0 | No |
| Persoonia glaucescens | Sh | McKenna | PhD thesis | *NA* | 2007 | AUS | 1 | 0 | No |
| Petrophile pulchella | Sh | Bradstock; O'Connell | Aust J Bot | 10.1111/j.1442-9993.1988.tb00999.x | 1988 | AUS | 1 | 0 | No |
| Phaseolus lunatus | H | Degreef; Baudoin; Rocha | Gen Res Crop Evol | 10.1023/A:1008623521755 | 1997 | CRI | 1 | 0 | No |
| Picris hieracioides | H | Klemow; Raynal | J Ecol | 10.2307/2259775 | 1985 | USA | 1 | 0 | No |
| Pinguicula alpina | H | Svennson; Carlsson; Karlsson; Nordell | J Ecol | 10.2307/2261662 | 1993 | SWE | 1 | 0 | No |
| Pinguicula villosa | H | Svennson; Carlsson; Karlsson; Nordell | J Ecol | 10.2307/2261662 | 1993 | SWE | 1 | 0 | No |
| Pinguicula vulgaris | H | Svennson; Carlsson; Karlsson; Nordell | J Ecol | 10.2307/2261662 | 1993 | SWE | 1 | 0 | No |
| Plantago media | H | Eriksson; Eriksson | J Veg Sci | 10.2307/3236803 | 2000 | SWE | 1 | 0 | No |
| Polemonium van-bruntiae | H | Bermingham | Plant Ecol | 10.1007/s11258-010-9762-5 | 2010 | USA | 1 | 0 | Yes |
| Potentilla anserina | H | Eriksson | J Ecol | 10.2307/2260610 | 1988 | SWE | 0 | 1 | Yes |
| Primula elatior | H | Jacquemyn; Brys | Ecology | 10.1890/07-1908.1 | 2008 | BEL | 1 | 0 | Yes |
| Pterocereus gaumeri | Su | Méndez; Duran; Olmsted | Biotrop | 10.1646/1601 | 2004 | MEX | 1 | 0 | No |
| Purshia subintegra | Sh | Maschinski; Baggs; Quintana-Ascencio; Menges | Cons Biol | 10.1111/j.1523-1739.2006.00272.x | 2006 | USA | 1 | 0 | No |
| Ranunculus acris | H | Sarukhan; Harper | J Ecol | 10.2307/2258643 | 1973 | GBR | 0 | 1 | Yes |
| Ranunculus bulbosus | H | Sarukhan; Harper | J Ecol | 10.2307/2258643 | 1973 | GBR | 1 | 0 | Yes |
| Ranunculus repens | H | Sarukhan; Harper | J Ecol | 10.2307/2258643 | 1973 | GBR | 0 | 1 | Yes |
| Rhus copallinum | Sh | Thaxton | PhD thesis | *NA* | 2003 | USA | 0 | 1 | Yes |
| Rubus praecox | H | Lambrecht-McDowell; Radosevich | Biol Inv | 10.1007/s10530-004-0870-9 | 2005 | USA | 0 | 1 | Yes |
| Rubus ursinus | H | Lambrecht-McDowell; Radosevich | Biol Inv | 10.1007/s10530-004-0870-9 | 2005 | USA | 0 | 1 | Yes |
| Sanicula europaea | H | Gustafsson; Ehrlén | Oikos | 10.1034/j.1600-0706.2003.11493.x | 2003 | SWE | 1 | 0 | Yes |
| Sarcocapnos baetica | H | Salinas; Suárez; Blanca | Can J Bot | 10.1139/b02-013 | 2002 | ESP | 1 | 0 | No |
| Sarcocapnos pulcherrima | H | Salinas; Suárez; Blanca | Can J Bot | 10.1139/b02-013 | 2002 | ESP | 1 | 0 | No |
| Sarracenia alata | H | Brewer | Am J Bot | 10.2307/3558336 | 2001 | USA | 0 | 1 | Yes |
| Saussurea medusa | H | Law; Salick; Knight | Plant Ecol | 10.1007/s11258-010-9761-6 | 2010 | CHN | 1 | 0 | No |
| Saxifraga cotyledon | H | Dinnetz; Nilsson | Plant Ecol | 10.1023/A:1015593311183 | 2002 | SWE | 0 | 1 | Yes |
| Scorzonera hispanica | H | Münzbergová | Folia Geobot | 10.1007/bf02806475 | 2006 | CZE | 1 | 0 | No |
| Silene acaulis | H | Morris; Doak | Am J Bot | *NA* | 1998 | USA | 1 | 0 | No |
| Silene spaldingii | H | Lesica; Crone | J Ecol | 10.1111/j.1365-2745.2007.01291.x | 2007 | USA | 1 | 0 | No |
| Sonchus pustulatus | H | Silva; Mejias; Garcia | Bas Appl Ecol | 10.1016/j.baae.2015.02.009 | 2015 | ESP | 1 | 0 | No |
| Stipa aristiglumis | H | Godfree; Lepschi; Rside; Bolger; Robertson; Marshall; Carnegie | Glob Change Biol | 10.1111/j.1365-2486.2010.02292.x | 2010 | AUS | 1 | 0 | Yes |
| Succisa pratensis | H | Jongejans; de Kroon | J Ecol | 10.1111/j.1365-2745.2005.01003.x | 2005 | NLD | 0 | 1 | Yes |
| Tetraneuris herbacea | H | Campbell; Husband | Heredity | 10.1038/sj.hdy.6800653 | 2005 | CAN | 0 | 1 | Yes |
| Thymus vulgaris | H | Iriondo; Albert; Giménez; Lozano; Escudero | Book | 978-84-8014-746-0 | 2009 | ESP | 1 | 0 | No |
|  | E | Mondragón; Ticktin | Cons Biol | 10.1111/j.1523-1739.2011.01691.x | 2011 | MEX | 1 | 0 | No |
| Tillandsia violacea | E | Mondragón; Ticktin | Cons Biol | *NA* | 2011 | MEX | 1 | 0 | Yes |
| Tragopogon pratensis | H | Milden; Eriksson | Ann Bot Fen | *NA* | 2007 | SWE | 1 | 0 | No |
| Trillium grandiflorum | H | Knight | Am J Bot | 10.3732/ajb.90.8.1207 | 2003 | USA | 1 | 0 | No |
| Ulex gallii | Sh | Stokes; Bullok;Watkinson | J Ecol | 10.1111/j.1365-2745.2004.00844.x | 2004 | GBR | 1 | 0 | No |
| Ulex minor | Sh | Stokes; Bullok;Watkinson | J Ecol | 10.1111/j.1365-2745.2004.00844.x | 2004 | GBR | 1 | 0 | No |
| Viburnum furcatum | Sh | Hara; Kanno; Hirabuki; Takehara | J Veg Sci | 10.1111/j.1654-1103.2004.tb02286.x | 2004 | JPN | 0 | 1 | Yes |
| Viola sagittata ovata | H | Solbrig; Sarandon; Bossert | Am Nat | 10.1086/284796 | 1988 | USA | 1 | 0 | No |
| Zea diploperennis | H | Sanchez-Velazquez; Ezcurra; Martinez-Ramos; Alvarez-Buylla; Lorente | J Ecol | 10.1046/j.1365-2745.2002.00702.x | 2002 | MEX | 1 | 0 | Yes |
| Acacia bilimekii | Sh | Jiménez-Lobato; Valverde | J Arid Env | 10.1016/j.jaridenv.2005.07.002 | 2006 | MEX | 1 | 0 | Yes |
| Actaea spicata | H | Fröborg; Eriksson | Can J Bot | 10.1139/B03-099 | 2003 | SWE | 1 | 0 | No |
| Agrimonia eupatoria | H | Kiviniemi | Plant Ecol | *NA* | 2002 | SWE | 1 | 0 | No |
| Agropyron cristatum | H | Hansen; Wilson | J Appl Ecol | 10.1111/j.1365-2664.2006.01145.x | 2006 | CAN | 1 | 0 | No |
| Allium tricoccum | H | Nault; Gagnon | J Ecol | 10.2307/2261228 | 1993 | CAN | 0 | 1 | Yes |
| Anemone patens | H | Williams; Crone | Ecology | 10.1890/0012-9658(2006)87[3200:TIOIGO]2.0.CO;2 | 2006 | CAN | 1 | 0 | No |
| Anthericum ramosum | H | Černá; Münzbergová | PLoS ONE | 10.1371/journal.pone.0075563 | 2013 | CZE | 0 | 1 | Yes |
